# Parallelisation strategies for agent based simulation of immune systems

**DOI:** 10.1186/s12859-019-3181-y

**Published:** 2019-12-10

**Authors:** Mozhgan Kabiri Chimeh, Peter Heywood, Marzio Pennisi, Francesco Pappalardo, Paul Richmond

**Affiliations:** 1 Department of Computer Science, University of Sheffield, Sheffield, UK; 2 Department of Mathematics and Computer Science, University of Catania, Catania, Italy; 3 Department of Drug Sciences, University of Catania, Catania, Italy

**Keywords:** Agent based modeling, GPGPU, High-performance computing, Cellular modelling, Computational modelling, Parallel simulation, FLAME GPU

## Abstract

**Background:**

In recent years, the study of immune response behaviour using bottom up approach, Agent Based Modeling (ABM), has attracted considerable efforts. The ABM approach is a very common technique in the biological domain due to high demand for a large scale analysis tools for the collection and interpretation of information to solve biological problems. Simulating massive multi-agent systems (i.e. simulations containing a large number of agents/entities) requires major computational effort which is only achievable through the use of parallel computing approaches.

**Results:**

This paper explores different approaches to parallelising the key component of biological and immune system models within an ABM model: pairwise interactions. The focus of this paper is on the performance and algorithmic design choices of cell interactions in continuous and discrete space where agents/entities are competing to interact with one another within a parallel environment.

**Conclusions:**

Our performance results demonstrate the applicability of these methods to a broader class of biological systems exhibiting typical cell to cell interactions. The advantage and disadvantage of each implementation is discussed showing each can be used as the basis for developing complete immune system models on parallel hardware.

## Background

The immune system comprises various biological structure and processes. Immune system models are a form of a complex biological system model which consist of a large number of agents (cells) communicating indirectly through diffusion of chemical substances or directly through connection of chemical receptors [1]. Due to the variance of type of interactions between various cells in a large-scale model, studying such system is challenging. Generally, to study and investigate biological systems, a hybrid approach that is the integration of experimental and computational research, is required. This hybrid approach has helped shaping novel hypotheses in research. In-silico experiments, a.k.a simulation, attempts to capture the dynamics of the system as an alternative to in-vitro/in-vivo for studying biological systems. With the hybrid approach, experiments that are not easily achievable in a laboratory are viable [2, 3].

### Agent based method

Modelling and simulation has been used by researchers in various scientific domains as a tool to better understand and predict the behaviour of a system. Based on the characteristics of the model, a system can be represented using different design methods. A top-down approach consisting of sets of equations can be used to model system level behaviour; or alternatively a bottom-up approach can be used where individuals within the system are modelled as agents. By using a top-down approach, it is possible to model large-scale systems of population dynamics (large number of entities). However, this approach ignores individual interactions as it approximates behaviour at the *macro*scopic level. The bottom-up approach uses a *micro*scopic level of modelling, where individual entities (agents) and interactions are described and then simulated to observe system level behaviour.

Agent Based Models (ABMs) and Multi-Agent Systems (MASs) are terms often used synonymously as techniques used to describe a model of a complex system. Both ABMs and MASs can be simulated to allow the non-linear behaviours of the complex systems to be studied [4, 5]. In other words, they are techniques for representing and describing an environment containing a number of agents (self contained entities) and the set of rules describing their behaviours and how they interact with each another and with the environment. In the context of an ABM, an agent can represent an individual or a collection of entities. Agent Based Simulations (ABSs) are the execution of an implementation of an ABM, for the purpose of studying the whole system. The ABM method provides a natural approach to modelling, where relatively simple behaviours and interactions are described, but more-complex behaviours may emerge during simulation. This makes the ABM approach suitable for the description of biological simulations, by describing the biological properties and behaviours of entities such as cells. It can then be simulated in order to study the complex and dynamic interactions within the biological environment. The ABM method is the most used bottom-up approach in immunology, as it describes the immunological process with higher accuracy than top-down approaches which may exhibit rough approximations [2]. With stochastic ABSs, the behaviour of large numbers of simple individuals can be aggregated over individual or multiple simulations to capture system-level behaviours [6].

### ABM modelling of cell-cell interactions

Within ABM different levels of abstraction can be applied. Agents such as cells may be modelled as points in continuous space where agents are modelled as particles moving with Brownian motion and interactions only occur based on spatial proximity and factors such as affinity. Alternatively, agents may exist within discrete spatial areas, or represent quantities of chemicals and cells at a discrete spatial location, which may be arranged in a regular structures (e.g. as a square or hexagonal lattice). Hybrid approaches where discrete spatial areas perform reaction diffusion modelling but have well mixed collections of directly interacting individual cells with Monte Carlo (pairwise interaction) are also common within immune system and more general biological modelling.

### Simulating ABM on GPUs

Compared to the top-down approach, simulating a complex system such as biological cellular system using ABM technique is computationally expensive. Increasing the scale of the model to achieve natural-scale simulations places additional computational burden which can impede discovery through simulation. A feasible solution would be the use of parallel computing resources to address this requirement and achieve improved simulation times when scaling agent based models of complex biological systems.

Graphics Processing Units (GPUs) are specialised massively parallel processors containing thousands of arithmetic processing units that can be utilised to achieve significant acceleration for computationally intensive scientific applications. GPUs allow a personal computer to be transformed into a personal supercomputer, providing up to 16 Trillion Floating Point Operations per Second (TFLOPS) in single-precision using consumer-grade hardware (NVIDIA TITAN RTX). While GPUs are computationally powerful, utilising high level of parallel performance is a huge challenge for a programmer without considerable knowledge of data parallel algorithms, the underlying parallel architecture and optimisation techniques.

GPUs have been widely used in many scientific research domains to accelerate applications and showed significant computational performance improvements [7]. There are several domain specific studies that use GPUs to implement various complex multi-agent systems [1, 8–10]. In the majority of these cases GPUs have been used for simulating continuous or discrete space abstractions as well as hybrid approaches which are desirable for large scale immune systems simulations.

In our previous work [10], we demonstrated the implementation of hybrid space biological models with parallel collection-type pairwise interactions executing on a GPU architecture. The pairwise cell-cell interaction was implemented using a hybrid approach, where agents representing multiple individuals at discrete locations interact with individuals at the same location. This paper (an extended version of paper [10] presented at Computational Methods for the Immune System Function workshop) describes three different parallel implementation of the pairwise interaction model which is representative of an agent based immune system model in both discrete or continuous space. The paper compares performance characteristics of three parallel implementations for a simplified large scale biological cellular system through a case study of interacting cells which from the basis of many immune system models. Types of interactions between cells (agents) and governed rules in the immune system models makes the model complex enough to be used as a case study to show the viability of using GPUs for other cellular level biological system with the same type of interaction pattern and complex behaviours.

Within the context of this paper, the model is implemented using three differing levels of modelling abstraction within the Flexible Large Scale Agent Modelling Environment for the GPU (FLAME GPU) framework [11], a flexible ABM environment for large-scale simulations that enables modellers from diverse scientific domains such as economics, biology and social sciences to easily write agent based models targeting GPUs [12]. We extended our previous work by using different approaches describing pairwise interactions which can be applied more broadly to general cell-cell or cell-environment interactions within an immune system model.

Our case study model is inspired by existing work that was implemented by the Universal Immune System Simulator (UISS) framework [13]. The UISS framework models and simulates immune system related pathologies on Central Processing Units (CPUs) and previously we demonstrated the feasibility of applying GPUs to biological cellular model by implementing a very common and necessary biological cell behaviour in FLAME GPU [10]. The study showed the applicability of the technique to a broader class of multi-cell biological system. This paper explores different approaches of implementing the same biological cell behaviour in FLAME GPU. Results from this study shows the performance comparison of these approaches.

The rest of this paper is organised as follows: The “Related work” section surveys previous studies on the application of GPU in Agent Based Modelling simulation, specifically in the field of biological cell modelling. “Methods” section presents design considerations required for different parallel implementation of the model. The “Results and discussion” section, reports the results of and discusses our experimental evaluation. Finally, we draw our conclusions in the “Conclusion” section.

## Related work

An immune system is an example of a complex system comprising different types of interactions between a variety of cell types. There are various ways to model immune systems. The most common approach is the use mathematical equations such as ordinary differential equations (ODEs) and partial differential equations (PDEs) [14–16]. One can capture entity changes over time while the later can capture changes in both time and space but is complex to solve. The equations are sometimes mathematically sophisticated but the complexity of the model is based on the number of equations describing the model. Generally, they are designed to model specific aspects of the immune response and rely on global information. Therefore, these models have difficulty exhibit the fluctuations typically observed in immunology [17]. In other words, biological phenomena cannot be easily captured using mathematical representations [18, 19].

Agent based methods provide ways of representing the heterogeneity of the entities as well nonlinear interactions among agents [20–23]. In ABM, agents interact with the environment or other individuals in continuous or discrete space. Arbitrary complex knowledge can be captured with agents in ABM. There are various existing works on agent based immune system models implemented using different levels of abstractions (continuous space, continuum or hybrid). Agent based Artificial Immune System (AbAIS) [24] framework uses a hybrid architecture where heterogeneous agents evolve over a cellular automata environment. In this framework, agents are modelled using a genetic approach. CAFISS [25] models cell-cell interactions in a grid where each cell has a bit string. The scalability of the model using this approach is questionable due to the large overhead caused by the use of separate thread for each cell. Each immune system cell in this approach runs its own thread. Cell to cell communication is performed through events.

ImmSim [17] is a framework based on cellular automata where entities interact with other and diffuse through lattice site. In this mode, individuals consider possible interactions based on the given probability rule. The framework has been developed in APL2, which due to language constraints limits the scale of simulations executed. Later, parallel version of ImmSim, C-ImmSim [26] were developed with the focus on scalability and performance. C-ImmSim is an advanced immune system simulation based on ImmSim with added features that allows simulations at the cells and molecules levels. The framework exploits task parallelism on distributed computers to reduce simulation runtimes and enable larger-scale simulations.

ImmunoGrid [27] uses C-ImmSim as an underlying framework. It uses grid technologies which allows very large and complex simulation size matching a real size immune system through distributed computing. Simmune [28] is a framework to model cell-cell and cell-molecule interactions where similar to ImmSim, cells do not have states. Simulating complex and detailed interaction using Simmune framework is very computationally expensive. Sentinel [29] is another framework based on the principles of ImmSim with environment is divided to grids and individuals can move between locations.

Jacob, Litorco and Lee[22] presented a swarm agent based 3D model of immune system in continuous space using Breve simulation [30]. Agents move randomly in the continuous space and only interact with those within their local radius. The visualisation and continuous space approach impose constraints on the simulation size [31]. Generally, simulating large scale complex models is computationally expensive.A possible solution is the use of Graphic Processing Units (GPUs). GPUs have been used to accelerate scientific application and proven to achieve significant performance for computationally problematic cases. There are several studies on the application of GPUs to biological systems [32–35]. There are several existing works on parallel implementation of the immune system model simulation in continuous space [1, 9, 36]. PI-FLAME [36] is a GPU-accelerated viral infection response simulator using continuous space, which demonstrates up to 13x reduction in simulation runtime compared to a serial CPU based implementation.

Chimeh et al. [10], implemented a specific type of cell interactions known as pairwise interaction in immune system model (common in biological cellular level systems) employed heavily throughout UISS [13]; a universal immune system simulator framework. UISS is a hybrid simulator combining ODEs and ABM in a discrete environment. Pairwise agent interactions occur between agents within each discrete location, which are assumed to be well-mixed, following stochastic processes. The UISS simulator is implemented for serial execution on CPUs, but design decisions were made to improve computational and memory efficiency, I.e. the use of discrete space rather than continuous space. Implementing a fine-grained data-parallel version of the model is non-trivial due to the extensive use of pairwise interactions which require conflict resolution in a parallel environment. Using FLAME GPU to simulate the simplified model with only two cell agent types with pairwise interactions, Chimeh et al. demonstrated that the technique is computationally more efficient than the serial counterpart and demonstrated the addition of a novel atomic based approach for reproducing equivalent serial behaviour.

Moreover, recently, the thermostatted kinetic theory methods have been employed for the modelling of various complex systems, e.g: cancer and immune system competition, social systems. The method is the combination of the mathematical formalism of ODEs and PDEs and interaction driven modelling of the ABM[37–39].

## Methods

We implement a simplified version of the pairwise interaction that exists in human immune systems and almost all models of biological systems. An example of this interaction can be seen between B cells (an immune system cell type that is part of the adaptive immune system) and antigens, or between Antibodies and Antigens. Our model uses the FLAME GPU library to map our model description to GPU executable code. In this section we describe the FLAME GPU framework used for our implementations; the properties and behaviour of our simplified pairwise model; and provide implementation details of the three alternate methods of parallel implementation.

### FLAME GPU

Developed since 2008, FLAME GPU framework is a generalised large scale ABM framework that employs the parallel architecture of Graphic Processing Unit (GPU) to enable real time model interaction and visualisation. FLAME GPU abstracts away the complexity of the GPU architecture from the users (modellers) by providing a high-level modelling syntax, based on a formal state-machine representation. The software aims to allow modellers from any domain to write a model to target GPUs capable of simulating millions of interacting individuals without the need to obtain specialist knowledge typically required to effectively program GPU architectures.

**Fig. 1:**
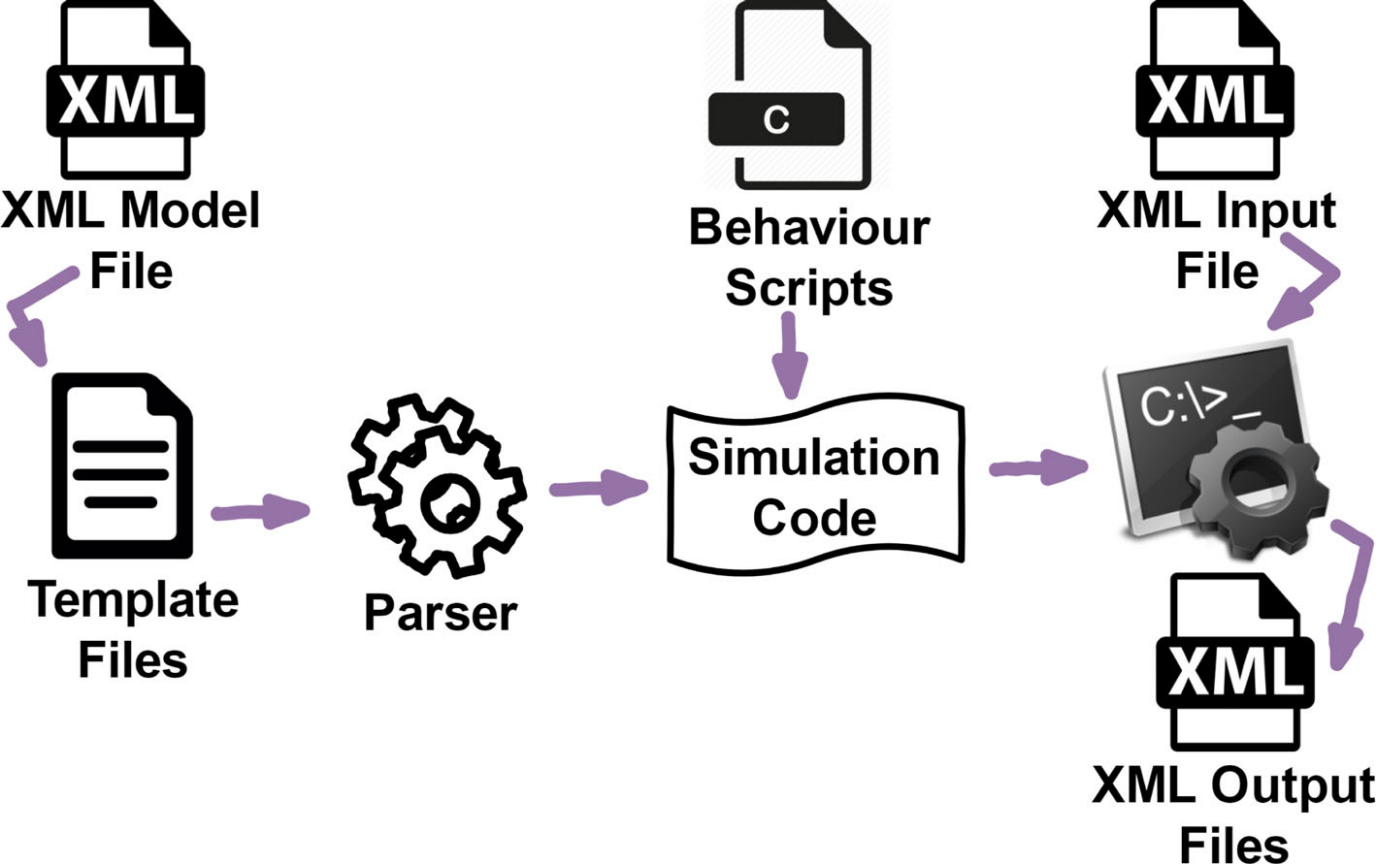
The FLAME GPU modelling process. An XSLT template processor translates a user defined XMML model into simulation code to be linked with the behavioural function scripts to produce a custom simulation executable

FLAME GPU is a template-based simulation environment that maps formal description of agents into simulation code. Agent representation is based on the concept of a communicating X-Machine where communication is performed via message lists. An overview of the features and capabilities of the FLAME GPU simulation platform has been demonstrated through an example in [12]. Figure 1 shows FLAME GPU code generation process which automatically translates a high level model description to optimised GPU code described in a series of code generation templates.

However, these types of interactions can be modelled and implemented in several way, each of which may have advantages and disadvantages. To extend this work we have created multiple implementations of a model characteristic of cellular immune system simulations, for a common interaction pattern.

### Model

A simplified model was designed to enable the evaluation of alternate highly parallel implementation strategies for pairwise interactions, typical of cellular immune system simulations. The pairwise interaction pattern is non-trivial in a highly-parallel environment with several alternate methods of implementation. For these types of model, the simulation environment can be modelled using either continuous space or using discrete space, and typically as a toroidal environment with wrapping in two axis.

The proposed model is designed to be suitable for implementation using either spatial modality. When using discrete space, the environment is assumed to be well-mixed within each discrete position. In continuous space, random sampling of uniform distributions can be used to achieved a well-mixed environment. For the purposes of this model and benchmark we will only consider a single location in the discrete environment. The model is designed for continuous time simulation, rather than discrete event simulation. The duration of a simulation is determined by the Simulation Length. The number of simulation iterations required will vary based on this and the length of time elapsed per simulation iteration.

The proposed model contains two populations A and B. These populations could represent different cells, proteins or other biological entities. For instance, the populations could represent B Cells and Antigens; or Antibodies and Antigens, in either continuous or discrete space. Some agents may be categorised based on certain properties. To account for this A agents have a value which represents the type of A. The number of types of A can be controlled through a model parameter.

Individuals from each population aim to interact with an individual population, in a mutually exclusive pairwise interaction. I.e. One A will interact with one B at a given point in time. The interactions are rate-limited, once an agent has interacted with a member of the other population it will not interact with another until a period of time has elapsed, referred to as a MACRO_TIMESTEP. To ensure reliable agent populations for benchmarking, once a pairwise interaction occurs agents simply record the event and progress. In a more realistic model, interactions would likely result in changes to the agent population, with existing agents being removed from the simulation, or new agents being created. Additionally, the interaction is probabilistic in nature, subject to implementation specific parameters. This enables each implementation to be calibrated to produce similar behaviour. For the purposes of calibration, each agent also records the number of interactions it achieves over the duration of the simulation. This can then be aggregated across the whole population at the end of the simulation.

Three implementations of this model are described: a continuous-space particle-like implementation; a discrete-space Monte Carlo style implementation; and a discrete-space collection-based implementation using agents which represent populations of multiple similar individuals.

### Implementation 1: particle

This fine-grained implementation of the model represents individual A and B agents as points within continuous toroidal space. Agents randomly move within the continuous environment, i.e. Brownian motion. Interactions between agents occur when in close proximity to a member of the other population. Additional stochasticity could be introduced through an additional probabilistic test, rather than solely relying on proximity. The rate of interaction is controlled through the maximum speed of movement of individuals and the interaction radius, controlled as model parameters.

The fine-grained nature of this implementation requires relatively short timesteps compared to alternate higher-level implementations. Each simulation iteration progresses time by a MICRO_TIMESTEP, where many MICRO_TIMESTEPs are required for a single MACRO_TIMESTEP to have occurred. This means that a larger number of simulation steps are required. As the individual simulation iterations are shorter than the MACRO_TIMESTEP described in the model, and interactions are rate-limited, agents become dormant after a successful interaction. While dormant, agents continue to move around the environment but are not involved in any potential interaction. Once an agent has been dormant for a full MACRO_TIMESTEP it once again becomes active.

**Fig. 2:**
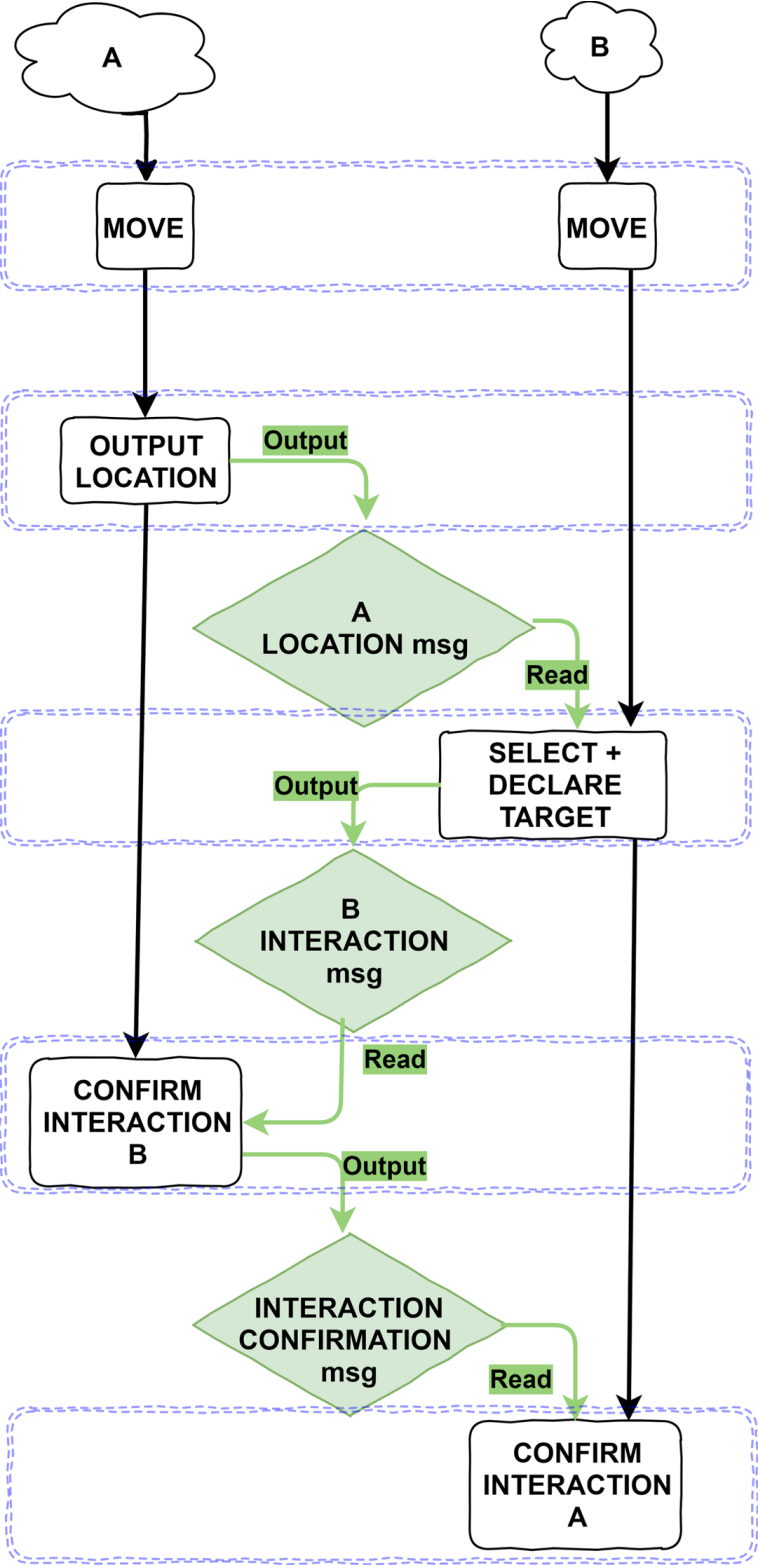
FLAME GPU state diagram for the particle implementation of pairwise cell interaction model. The diagram shows the order of agent functions (black rectangles) and interactions between agents via message lists (coloured rhombuses) through a single iteration of the main simulation loop. Within a layer, indicated by blue dashed boxes, functions may execute concurrently. In the first function layer A agents and B agents both execute their respective move behavioural function, where individuals move using Brownian motion. In the second layer, A agents execute the Output Location function, broadcasting their location within the simulation environment to the A Location message list. This message list is iterated by B agents in the Select + Declare Target function, where B agents select the A agent they wish to interact with and broadcast it into the B Interaction message list. In the fourth layer, A agents iterate the B Interaction message list, deciding which B interaction they will participate in and output a message as confirmation. Finally B agents iterate the confirmation messages to discover if they were successful, and if so they behave appropriately

Figure 2 shows the state machine employed within the FLAME GPU model, representing the process for a single iteration. At the start of each iteration Agents from both the A and B populations execute their respective MOVE function. This uses Random Number Generation (RNG) to calculate a new position for the individual. Additionally, any agents which are dormant will reduce the time until their next interaction can occur.

In the second layer, A agents output a message broadcasting their location in continuous space and other publicly visible properties required by B agents, such as their unique identifier and type. The message list produced is then iterated by each agent in the B population in the third function layer. Each B agent selects the closest member of the A population within the local interaction radius and outputs a message containing it’s intent.

Next, A agents iterate the interaction message list in the CONFIRM INTERACTION B agent function, to determine which member of the B population is the closest. Internal state of the A agent is then modified accordingly, and a final message is output, confirming the successful interaction to the relevant B agent. This message allows the B to also update it’s internal state, including the dormant status, in the final agent function CONFIRM INTERACTION A. This process is repeated at each simulation iteration.

### Implementation 2: Monte Carlo

This Monte Carlo style implementation uses a higher-level of abstraction than the previous particle-based implementation, which probabalistically approximates the interactions between agents at a discrete location. Individual A and B agents are assumed to be well-mixed within a site of the discrete environment. The environment would typically be a square or hexagonal toroidal lattice, but for the purposes of this paper only a single large lattice site is considered. A relatively large timestep is used for this implementation, the MACRO_TIMESTEP. A shorter timestep is not required due to the coarse nature of the simulation, which does not include finely-grained temporal behaviour. The probabilistic pairwise interaction is deterministically processed using a process of rank-generation, sorting and matching.

**Fig. 3:**
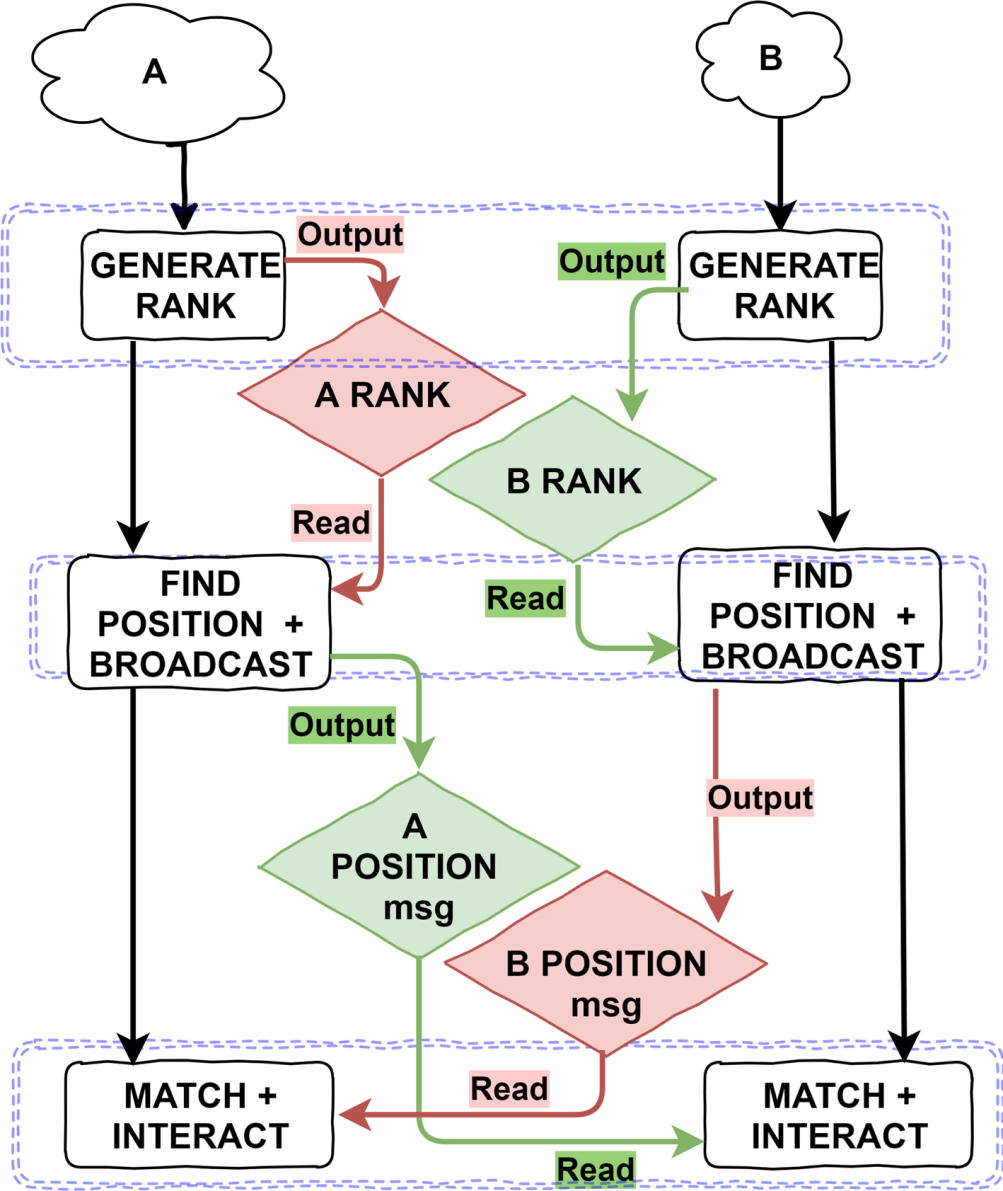
FLAME GPU state diagram for the Monte Carlo implementation of pairwise cell interaction model. The diagram shows the order of agent functions (black rectangles) and interactions between agents via message lists (coloured rhombuses) through a single iteration of the main simulation loop. Within a layer, indicated by blue dashed boxes, functions may execute concurrently. For the Monte Carlo implementation, A agents and B agents both execute their respective Generate Rank functions in the first layer. The ranks of each respective agent type are output in to respective message list, A Rank and B Rank. In the second layer, agents from each population iterate the message list from their own population to find the location within the rank-list, before broadcasting this information to the other type of agent via the relevant Find Position + Broadcast function. Finally, in the third layer, Agents iterate messages form the opposing population to determine if they are able to interact based on their position within their population and also information about the opposing population

Figure 3 shows the FLAME GPU implementation state-diagram for the Monte Carlo implementation. Initially, at the beginning of each iteration, agents from each population are assigned a unique rank within the respective A or B population, in the GENERATE_RANK method. These ranks are assigned randomly to different individuals per-simulation iteration, to avoid bias towards agents based on location within the list of agents. Each agent broadcasts their rank to a message list per population (along with other publicly visible information such as unique identifier).

Agents in each population then iterate messages from their own population, to find their own position within the rank list of that population. I.e. A agents iterate all A RANK messages, counting the number of individuals with a lower value rank (indicating a higher priority) than themselves. Counting the number of agents within the population with a lower rank improves the robustness of the model, by enabling the use of non-sequential integer ranks (in cases where agents may have been removed from the simulation) or non-integer rank values. The position within the population is then broadcast by each agent to a separate message list per population.

Finally, in the third layer, agents from each population iterate messages from the opposing population to find the individual within the other list at the same position within the rank list. If populations are different sizes some agents will not have a matching member of the other population. Agents with sufficiently low rank within the population, based on the parametrised interaction rate, are allowed to interact and modify their state accordingly. Additional variance could be introduced with a per-interaction probabilistic test.

### Implementation 3: collection agents

The Collection implementation does not directly map individual members of the Apopulation to individual agents. Instead, agents which represent a collection of similar individuals are used, referred to as AC agents. Individual agents are used to represent individual members of the B population, although collection-collection interactions would be viable. This approach is only applicable to models where a population of entities can be grouped into collections of similar individuals, and where the specific actions of individual members of the group are not important.

AC agents and B agents are modelled in discrete-space, i.e. a toroidal lattice with uniform mixing of agents per lattice site, although this implementation only considers a single lattice site. Each simulation iteration represents a larger MACRO_TIMESTEP, requiring fewer iterations than finer-grained implementations.

This approach is only suitable where the individuals from the A population can be categorised into a collection of similar agents, where the individual characteristics and behaviours are not important. I.e. members of the same type of antigen which exist in the same discrete location. AC agents have a variable, quantity containing the number of individual As of that type represented by the AC agent. In this case, the probabilistic interaction is controlled by an interaction rate for a given B to interact with an A as a parameter.

**Fig. 4:**
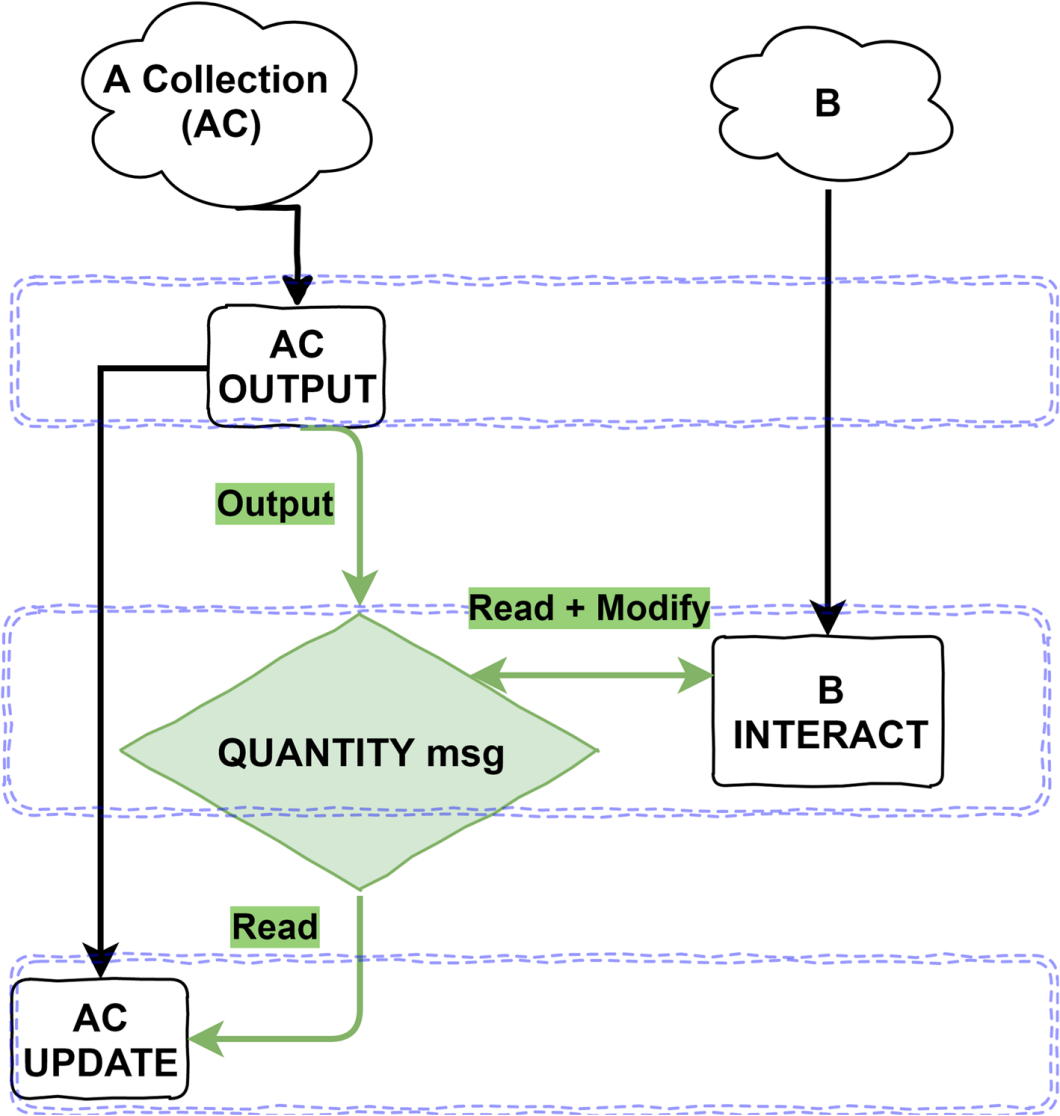
FLAME GPU state diagram for the collection implementation of pairwise cell interaction model. The diagram shows the order of agent functions (black rectangles) and interactions between agents via message lists (coloured rhombuses) through a single iteration of the main simulation loop. Within a layer, indicated by blue dashed boxes, functions may execute concurrently. In the first function layer, AC agents output their publicly visible information to the quantity message list. This information includes the type and quantity of A which the AC agent represents. B agents then execute the B Interact function in the second layer. Each B agent iterates the message list, attempting to interact with AC agents where appropriate. This depends on their being sufficient quantity for an interaction to occur, implemented using atomic operations on the message data. Finally, in the third layer once all possible interactions have occurred, AC agents iterate the modified quantity message list to find the message they originally output, and update their local data such as quantity to match the modified message data

Figure 4 shows the implementation state-diagram. In the first function layer, AC agents output their publicly visible properties to a message list, including the number of individuals represented and the category or type of A. B agents then iterate the quantity messages, and probabilistically attempt to interact with a single individual from the collection. This is implemented using an atomic operation, an operation which is guaranteed to occur without the impact of a concurrent thread. If the probability test passes within the B INTERACT method, the B agent attempts to atomically subtract 1 from the quantity value stored in the message. If this succeeds then the interaction is considered successful, otherwise, if the atomic operation could not reduce the quantity or the agent did not pass the probability test, then the B agent will attempt to interact with another A represented by a different AC. In the third and final layer, AC agents re-read the message they originally output, to update their own state regarding the number of remaining A represented after interactions have occurred. The quantity value is then reset for the next iteration to ensure a consistent population for benchmarking purposes.

The use of the atomic operation prevents potential concurrency issues, but also introduces some factors to be considered. Atomic operations can prevent race-conditions - where multiple agents (threads) would potentially believe they had successfully reduced the quantity value, but in fact the limited quantity had already been exhausted. In order to reduce serialisation within the implementation and the number of atomic operations performed, rather than attempting a probability test per A represented it is preferable to perform a single test per B-AC interaction. This requires an alternate probability threshold, calculated using the binomial probability. Furthermore, the use of atomic operations in a highly parallel environment introduces non-determinism, subsequent runs of the same simulation may not produce the same behaviour. In many-core processors such as GPUs, the order in which threads execute is not guaranteed. This results in the atomic operations potentially being issued in different orders, and therefore different agents may succeed in different interactions on subsequent simulations. However, as many simulations are required due to the highly stochastic nature of the model this variation should be absorbed during aggregation of many simulation runs.

### Implementation calibration

Although we are not calibrating a model to real-world data, we calibrated the three implementations to produce the same aggregate behaviour for a fair system level performance comparison. This was manually performed at a single scale, targeting an average number of interactions per MACRO_TIMESTEP of 512 for simulations containing 1024 Bs, 1024 As and 32 types of A. The simulation duration was set to 1000, with a MACRO_TIMESTEP of 10, and a MICRO_TIMESTEP of 0.1. Table 1 shows the model parameters used for each implementation, and the observed aggregate interaction rate.

**Table 1:** Calibration Parameters

Parameter	Particle	Monte Carlo	Collection
MACRO_TIMESTEP	10	10	10
MICRO_TIMESTEP	0.1		
Maximum Speed (normalised environment)	0.01		
Interaction Radius (normalised environment)	0.0177		
Interaction Probability		0.00068	0.00068
Interactions per MACRO_TIMESTEP	511.6	513.0	511.0

Contains the calibration parameters and resulting interaction rate for the simple cross-calibration

## Results and discussion

To evaluate the runtime performance of each implementation, a range of simulations were carried out varying the populations scales and therefore agent density. The number of As and number of Bs represented by the simulation were varied for all implementations, between 2^8^ to 2^19^. Additionally, the number of types of A and therefore AC population were also varied for the collection implementation, with values of 128, 512, 1024, 4096, 16384 and 65536. Each simulation was repeated 3 times, using 3 seed values for RNG, and the runtime of each repetitions is recorded. All benchmarks were performed using on a Ubuntu 16.04 workstation containing an Intel Core i7-6850k and NVIDIA TITAN V GPU running Ubuntu 16.04 and driver Nvidia 418.40. Applications were built using a modified version of FLAME GPU 1.5, GCC 7 and CUDA 10.0, optimised for the Nvidia Volta GPU architecture (SM_70).

**Fig. 5:**
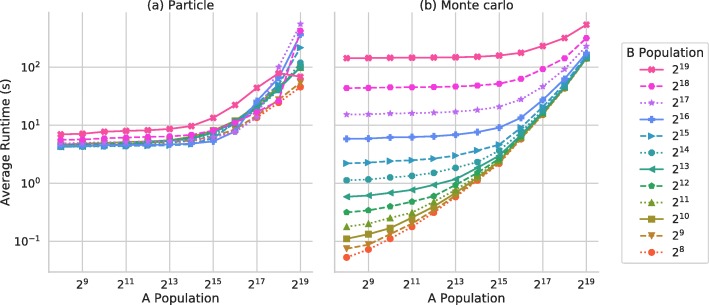
Average simulation runtime for the particle and Monte Carlo implementations. Average simulation run time against population size the **a** particle and **b** Monte Carlo model implementations. Average run time in seconds across 3 repetitions of 3 random seeds as the populations of A agents and B agents are scaled

**Fig. 6:**
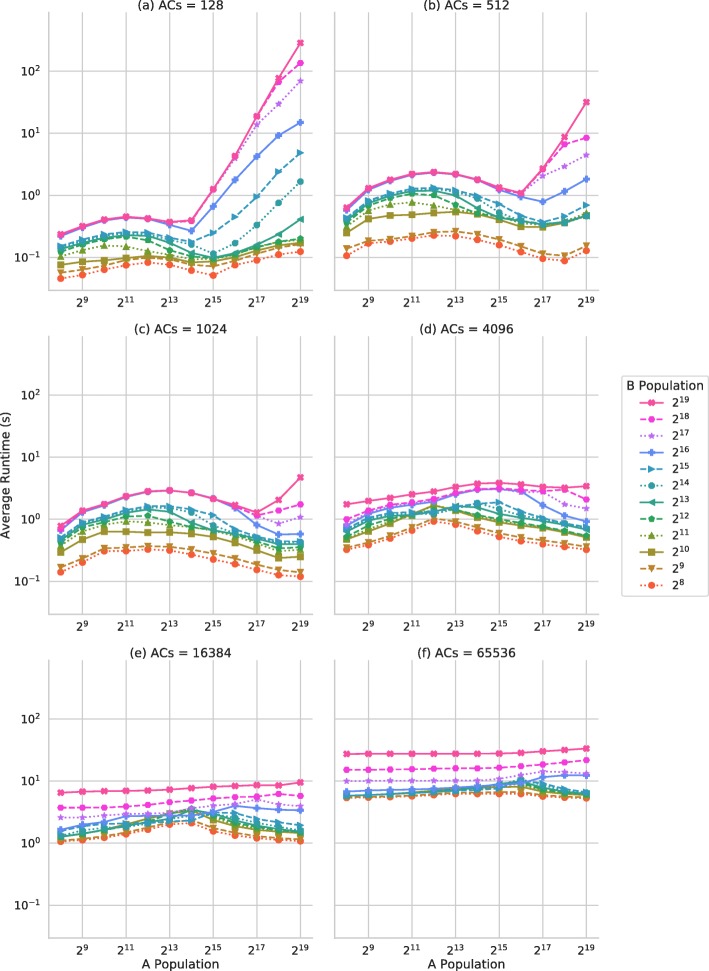
Average simulation runtime for the collection implementation. Average simulation run time for the collection implementation of the model. Average run time in seconds across 3 repetitions of 3 random seeds as the B agent population and the total number of A represented and the number of AC agents are varied (sub figures (**a**) to (**f**))

Figure 5a and b show the average simulation runtime as the A population and B population are varied for the particle and Monte Carlo implementations respectively. Figure 6 shows the average simulation runtime for the collection implementation, as the total number of As, Bs and the number of types of AC are varied.

The particle-based implementation generally shows the longest simulation runtimes of all the implementations. This can mainly be attributed to the greater number of iterations required than the alternate implementations and the fine-grained movement of individuals, the implementation is less work-efficient. On the other hand, the implementations are relatively simple and intuitive to understand, with simple logic to determine successful pairwise interactions. At smaller scales, up to around 2^14^As or Bs there is only a minimal increase in runtime as the population increases. At this scale of simulation the TITAN V GPU is not being sufficiently utilised to mask the overhead costs and latency of GPU computing. Once the agent populations become larger and the device achieves high levels of utilisation are achieved, simulation runtimes increase at a greater rate. The main increase in runtime above 2^14^ agents can be attributed to the cost of iterating larger message lists, as the density of the simulation environment increases. In this case only local messages are iterated, reducing the number of messages to be iterated by each agent. Additionally, the stochastic nature of these simulations which rely on RNG can have a measurable impact on the performance of the simulations. Each simulation case was repeated 3 times with the same seed, but also using 3 different RNG seeds to account for this. The seed alone accounted for a variance of ±1.7*%* of the mean runtime for a given simulation configuration.

The Monte Carlo style implementation shows the broadest range of simulation runtime. Small scale simulations run quickly compared to the particle based implementation, as fewer simulation iterations are required. As the populations are increased however simulations become much slower. In part, this is due to the relatively expensive generation of unique rank values per iteration, which becomes more costly as the number of rank values to be scattered grows. This accounts for the consistent simulation runtimes for simulations with 2^19^A agents or B agents, which are some of the slowest of all 3 implementations. The size of message lists to be iterated also contributes to the poor performance at large scales.

The collection implementation which uses the AC collection agents exhibits different performance characteristics dependent upon the number of AC agents used, shown by facets (a) to (f) in Fig. 6. Typically this implementation shows better performance than the particle based simulator, due to the reduced number of iterations required, and better performance than the Monte Carlo based implementation for most AC populations. For low numbers of AC agents such as in Fig. 6a and b with low populations of B agents performance is consistent as the number of As represented increases. However, for larger B populations and larger quantities of A, performance degrades significantly. This is due to *atomic contention*. When large numbers of atomic operations are issued to the same memory address concurrently, parallelism is reduced as the atomic operations must be resolved in serial, resulting in an increased runtime. For larger numbers of AC agents, the average quantity is reduced, resulting in a smaller loss of performance due to atomic contention; although the total runtime increases as message lists are larger. Additionally, simulations with fewer AC individuals do not make good use of the highly parallel GPU, which may have a significant impact on simulator runtime in more realistic models with more complex interaction behaviours.

For larger populations of AC agents, such as Fig. 6f, performance is relatively consistent regardless of the number of A represented, as atomic contention is less of an issue, and the number of agents and therefore threads is consistent. Larger populations of B show a reduction in performance, as the number of threads increases and over-saturate the GPU, resulting in serialisation.

Each implementation has advantages and disadvantages, with respect to both modelling and simulator performance, with no clear optimal implementation approach for all use-cases. The particle-style implementation has poor work-efficiency and therefore relatively poor performance compared to the alternate approaches, but the modelling approach may be advantageous due to its intuitive nature and fine-grained data capture. The Monte Carlo style implementation has good performance characteristics for smaller models, but performance does not scale well with problem size, showing the broadest range of simulation runtimes of the three implementations. This approach does have advantages regarding the modelling approach, with a high degree of reproducibility, but with coarser data-capture. Lastly, the collection implementation shows the best performance of the three approaches for larger-scale simulations, with the best performance- scaling behaviour. However, when only a small number of collection agents are used to represent a larger overall population, significant performance degradation is observed due to both low device-utilisation and high levels of atomic contention and therefore serialisation. Additionally, the collection style is more memory-efficient than the other implementations for the same population, enabling larger simulations without the need for a multi-GPU or multi-node simulation.

Both the model and the set of benchmarks have several limitations which would benefit from further exploration. The abstract model only considers a single site in discrete space. In less-abstract models the environment would typically be represented by a square or hexagonal lattice, with toroidal wrapping. Essentially we have been varying the density of the simulations with scale. It would be interesting to evaluate the performance impact of scaling populations with a fixed average initial density to evaluate scaling towards natural size simulations, by modelling multiple lattice sites. Ideally this would involved additional agent behaviours to mimic the movement between sites in the environment. This should show better performance scaling than observed by increasing density, as message lists can be optimised, reducing the number of messages iterated by each agent.

The approach could also be extended to interactions involving more than two agents, however once more individuals are involved it will become more challenging to find an approach which does not add further serialisation or synchronisation steps which should be minimised in a many-core parallel environment to maintain performance.

Additionally, the benchmark model does not vary the populations as the simulations progress. This is useful for understanding the performance impact of the modelling approach, but is not representative of a more realistic model.

Lastly, RNG can have a significant impact on the performance of some models, such as the particle implementation. The total impact of random number generation is relatively small for this abstract model, but with more realistic models where probabilistic interactions result in the creation or death of agents the effects of RNG will be amplified.

## Conclusion

This paper is the extended version of work published in [10] which proved the feasibility of applying GPU to implement a hybrid pairwise interaction model representative of an agent based immune system model. Previous paper showed using FLAME GPU to simulate the simplified model with only two cell agent types with pairwise interactions using the collection approach, we demonstrated that the technique is computationally more efficient than the serial counterpart.

There are various ways to model this type of interactions. This paper explored three different parallel implementation of this specific type of cell interactions (in both discrete and continuous space) known as pairwise that is very common in biological cellular level systems.

Our results showed that each implementation has its own advantages and disadvantages with respect to simulator performance and model characteristics; and there is no universally optimal solution. Based on our experimental results, among three different form of implementations presented in this paper, the Collection style implementation offers the highest levels of performance for large-scale simulations. This approach would be well suited for models where agents can be grouped into collections of similar agents and capturing individual behaviours is not required. For smaller scale simulation models where agents cannot be grouped into collection of similar individuals, the MC implementation offers better performance. Due to due to a greater number of higher-precision timesteps required for the same simulation duration, the Particle implementation performs relatively poor compared to the other two approaches. However, this method offers more detailed microscopic behaviour and is suitable for use in GPU simulations.

## Supplementary information

**Additional file 1** CSV containing raw benchmarking data for each implementation. Contains simulation configuration and runtime for each individual run.

## Data Availability

All data generated during this study are included in the Additional file 1.

## Abbreviations

ABM: Agent based model
ABS: Agent based simulation
CPU: Central processing unit
FLAME GPU: Flexible large scale agent modelling environment for the GPU
GPU: Graphics processing unit
MAS: Multi-agent system
ODE: ordinary differential equation
PDE: partial differential equation
RNG: Random number generation
TFLOPS: Trillion floating point operations per second
UISS: Universal immune system simulator
